# Supplementary material for: An integrative molecular map of pediatric B-cell precursor acute lymphoblastic leukemia
Source: Commun Med (Lond). 2026 Apr 11;6:222. doi: 10.1038/s43856-026-01568-9 (PMC13079846; doi:10.1038/s43856-026-01568-9)
Supplement: Supplementary file 2 — Supplementary Information [file 43856_2026_1568_MOESM2_ESM.pdf]

Supplementary Information

An integrative molecular map of pediatric B-cell precursor acute lymphoblastic leukemia

Olga Krali[1,2], Anna Pia Enblad[1,2,3], Julia Sulyaeva[1,2], Dea Gogishvili[1,2], Anders Lundmark[1,2], Arja Harila[3], Claes Andersson[1], Tom Erkers[4,5], Merja Heinäniemi[6], Gudmar Lönnerholm[3], and Jessica Nordlund[1,2]*

1. Department of Medical Sciences, Uppsala University, Uppsala, Sweden
2. SciLifeLab, Uppsala University, Uppsala, Sweden
3. Department of Women's and Children's Health, Uppsala University, Uppsala, Sweden
4. Department of Oncology-Pathology, Karolinska Institutet, Stockholm, Sweden
5. SciLifeLab, Stockholm, Sweden
6. Institute of Biomedicine, School of Medicine, University of Eastern Finland, Kuopio, Finland

*Corresponding author:

Dr. Jessica Nordlund

Box 1432, BMC

75144 Uppsala, Sweden

Telephone: +46 704250806

Email: jessica.nordlund@medsci.uu.se

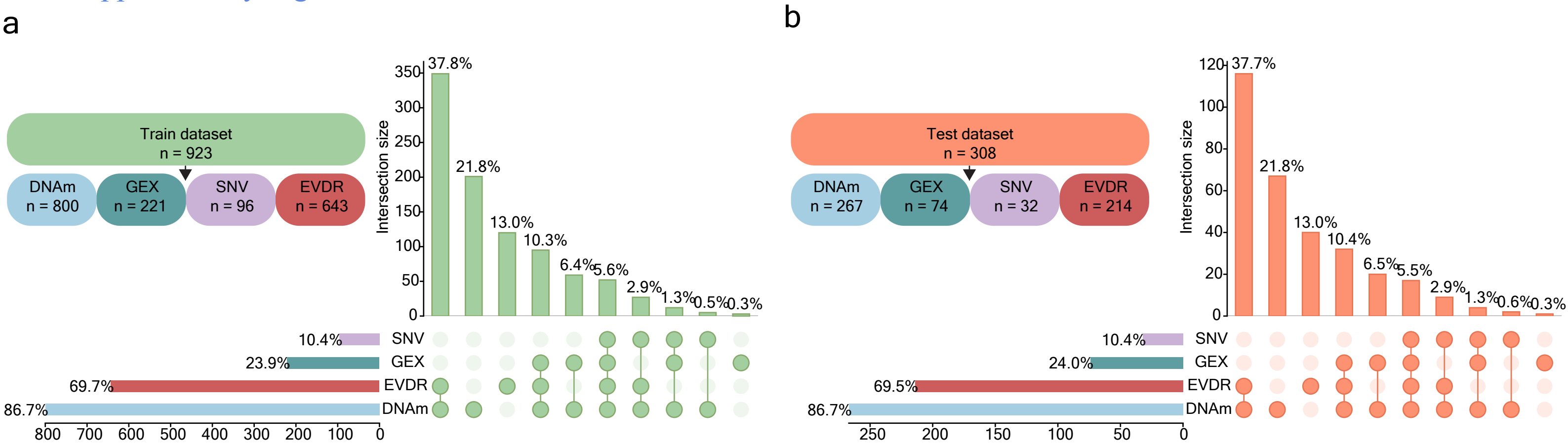

Fig. S1. Modality distribution on the training (n = 923) and test (n = 308) datasets. Percentage-wise sample distribution across each modality and upset plot demonstrating the matched samples between two, three, all or none of the modalities in the (a) train and (b) test datasets.

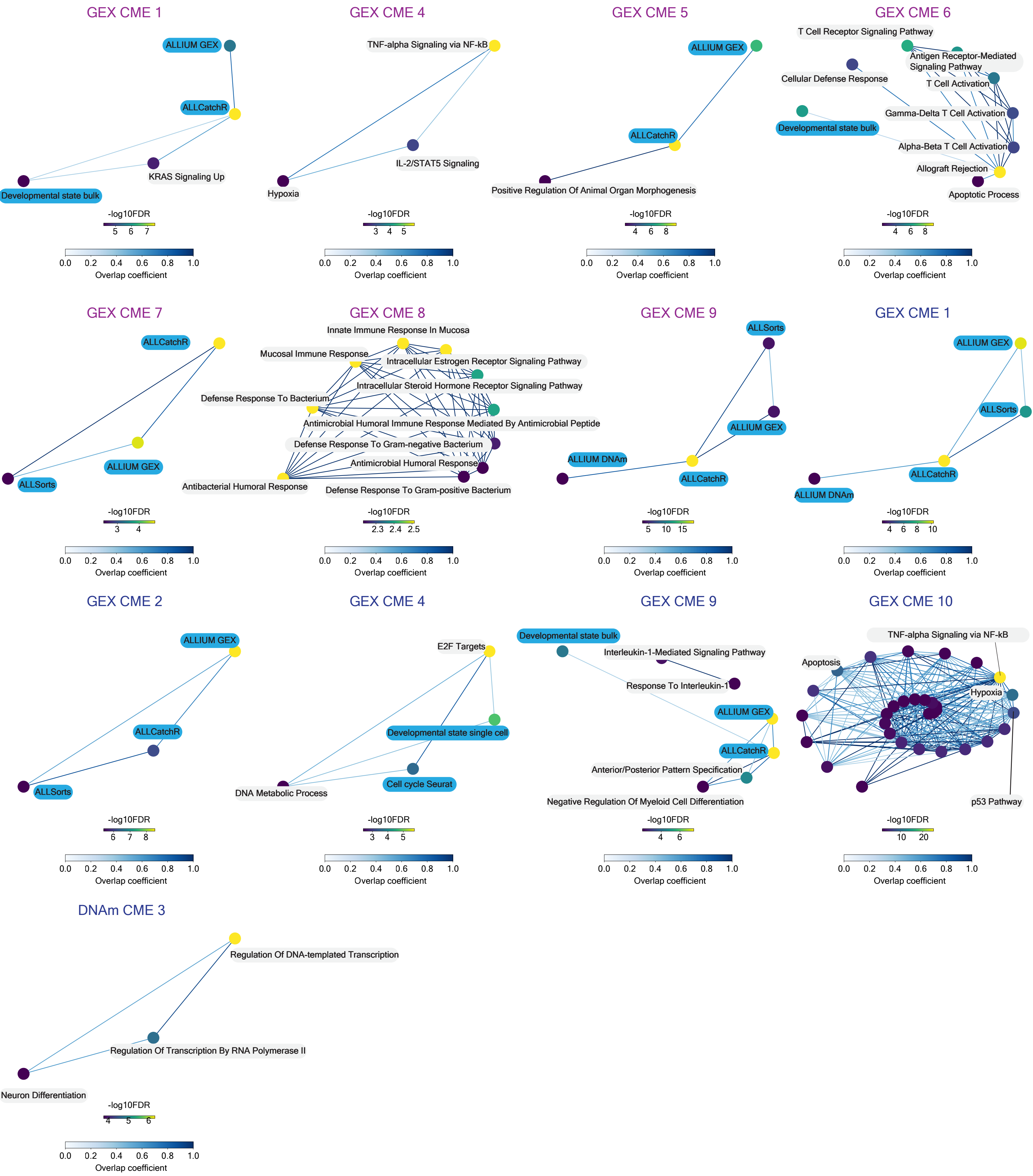

Fig. S2. Networks of the significantly enriched pathways (FDR <0.01) across CMEs. The CME-specific pathways were either enriched in genes (GEX) or CpG sites (DNAm) with positive (magenta) or negative weights (blue). Pathways derived from the Gene Ontology biological process or the molecular signatures hallmark databases are color-coded with gray boxes, whereas the custom gene lists with cyan. The pathway nodes are color-coded based on their -log$_{10}$FDR score and edge connectivity is color-coded based on the overlap coefficient.

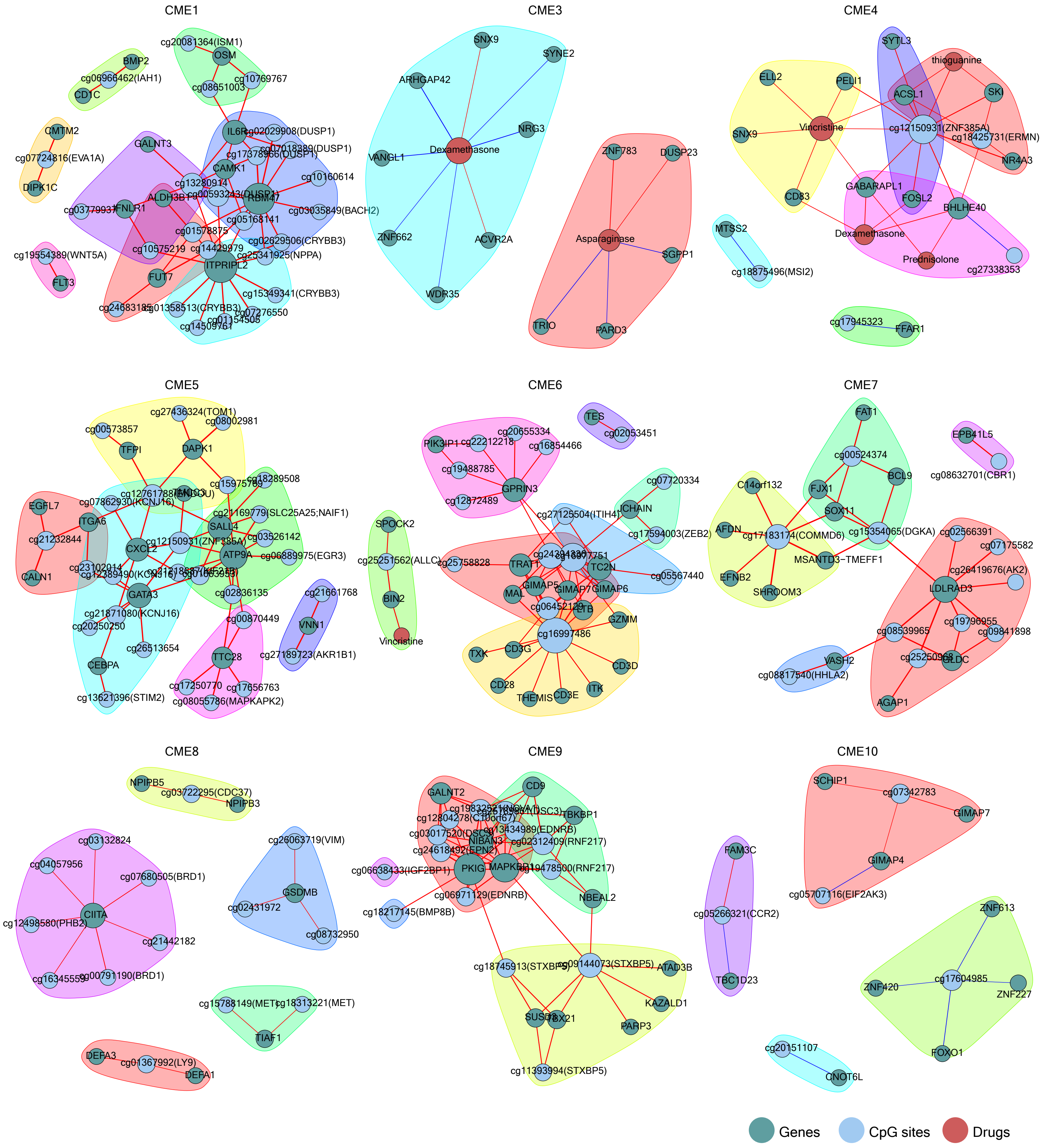

Fig. S3. Networks for each cross-modal element (CME) for features with positive weights for the training dataset (n = 923). Positive correlations (edges) are color-coded with red while negative with blue color.

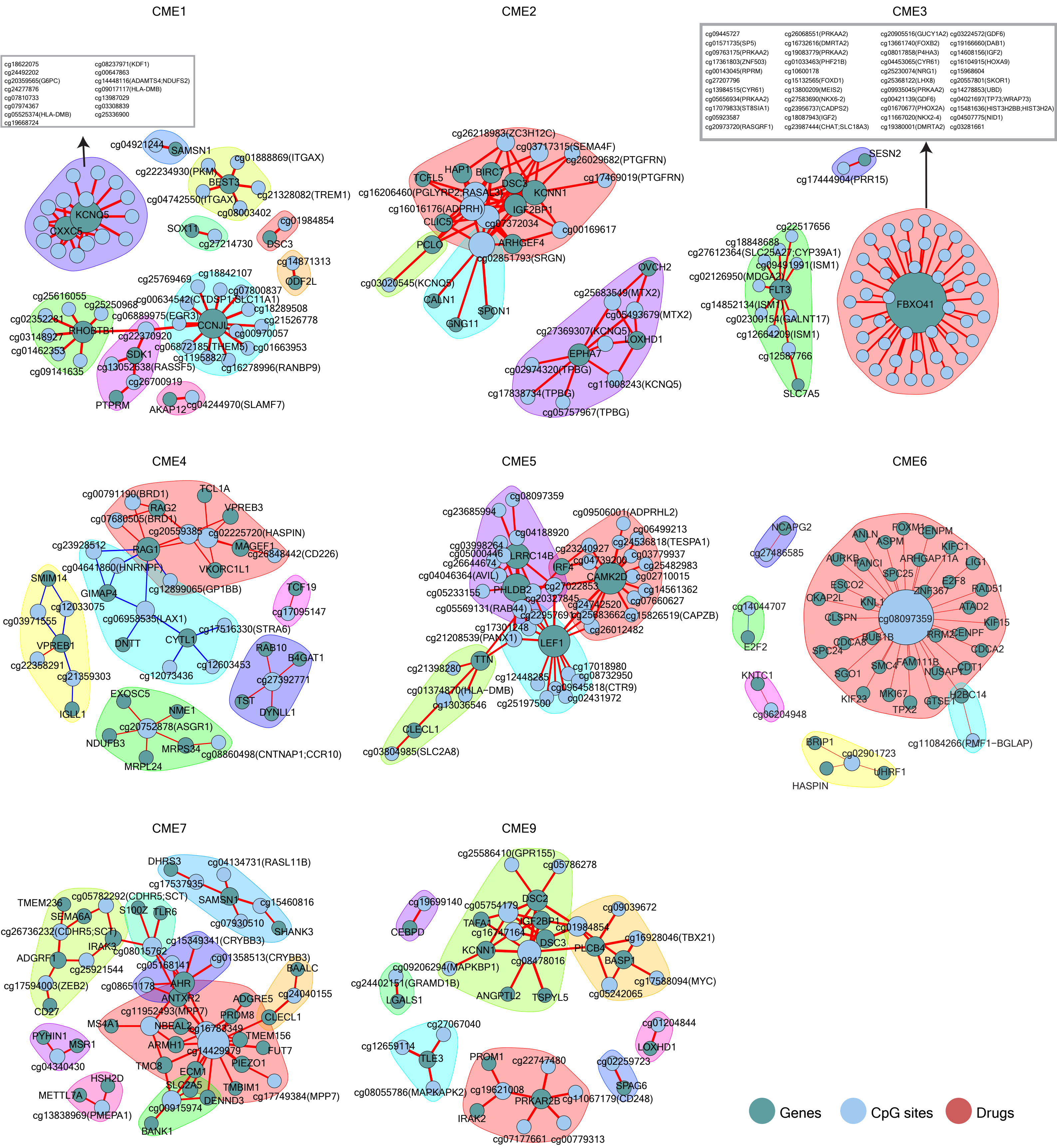

Fig. S4. Networks for each cross-modal element (CME) for features with negative weights for the training dataset (n = 923). Positive correlations (edges) are color-coded with red while negative with blue color.

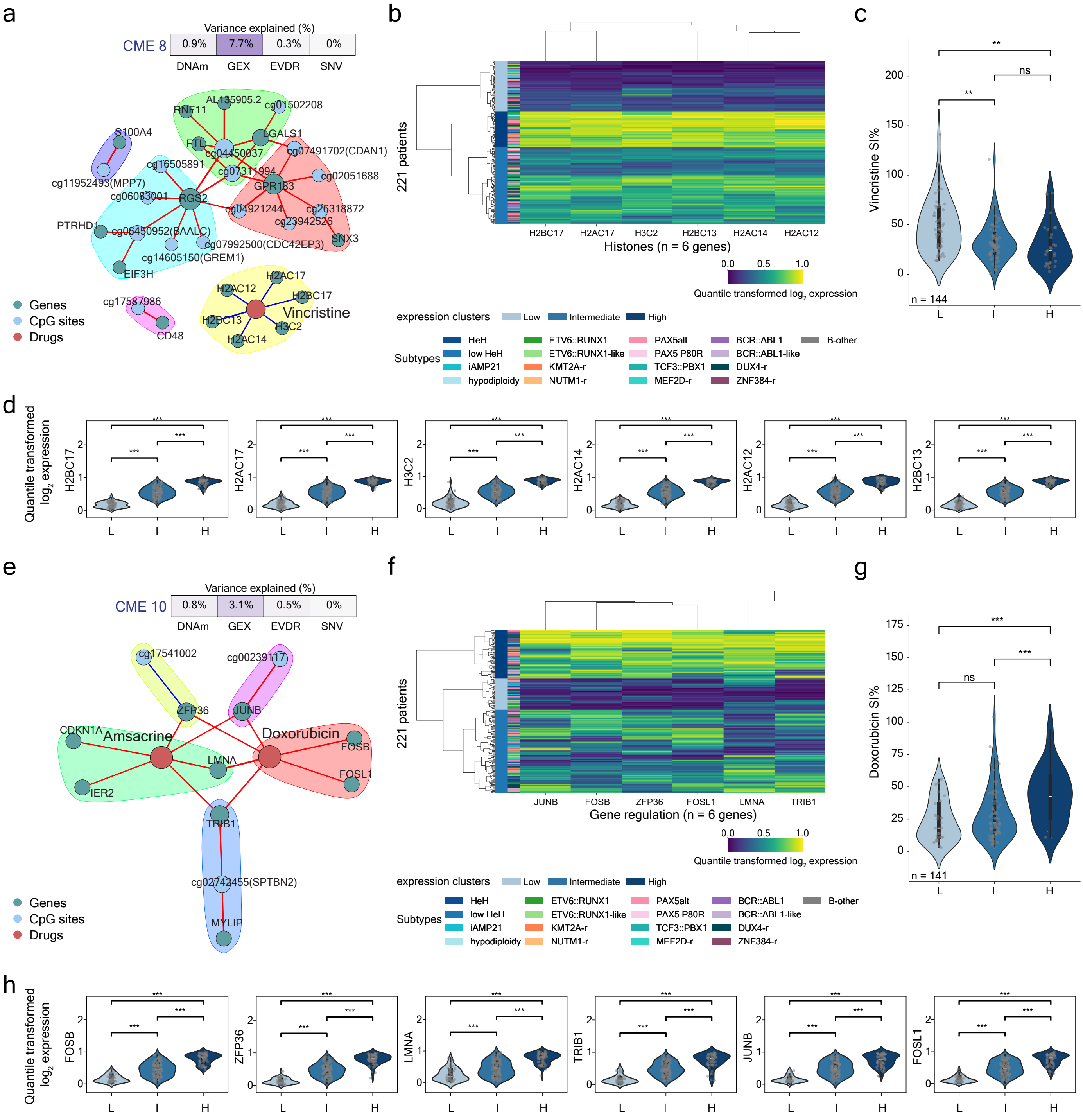

Fig. S5. Inter-modal networks and correlations in the train dataset (n = 923 samples). a) Inter-modal networks for features with negative weights and absolute rho >0.35 in CME 8. The variance explained by CME 8 is shown at the top of the panel. Positive correlations are denoted with red and negative in blue. The size of each correlation (edge) is based on the correlation value, while the size of each circle (vertex) on the number of connections. b) Heatmap of expression levels of the six genes annotated to the vincristine hub (x-axis) across 221 patients color coded by expression cluster (low-high, y-axis) ordered by unsupervised hierarchical clustering. The gene expression clusters and the molecular subtypes are shown as annotation bars on the y-axis. c) Violin plot of SI% after vincristine treatment (y-axis) across the three gene expression clusters (x-axis). d) Expression (y-axis) for the histone genes across the three expression groups (x-axis). e) Inter-modal networks for features with negative weights and absolute rho >0.3 in CME 10. The variance explained by CME 10 is noted at the top of the panel. Positive correlations are denoted with red and negative in blue. The size of each correlation (edge) is based on the correlation value, while the size of each circle (vertex) on the number of connections. f) Heatmap of genes expression levels of six genes in the doxorubicin hub across 221 patients color coded by gene expression groups (low-high, y-axis) ordered by unsupervised hierarchical clustering. The gene expression clusters and the molecular subtypes are shown as annotation bars on the y-axis. g) Violin plot of SI% after doxorubicin treatment (y-axis) across the three gene expression groups (x-axis). h) Expression (y-axis) for gene regulation genes across the three expression groups (x-axis). Dunn's test Benjamini-Hochberg (BH) adjusted p-values: *** < 0.001, ** < 0.01, * <0.05, ns: non-significant. L: low, I: intermediate, H: high.
