# Supplementary material for: An integrative molecular map of pediatric B-cell precursor acute lymphoblastic leukemia
Source: Commun Med (Lond). 2026 Apr 11;6:222. doi: 10.1038/s43856-026-01568-9 (PMC13079846; doi:10.1038/s43856-026-01568-9)
Supplement: Supplementary file 3 — Description of Additional Supplementary Files [file 43856_2026_1568_MOESM3_ESM.pdf]

Description of Additional Supplementary Files

File name - **Supplementary Data S1.**

File description - Drug concentrations for the ten drugs used for the fluorometric microculture cytotoxicity assay (FMCA) and the total number of patients tested per drug.

File name - **Supplementary Data S2.**

File description - Data availability across modality and dataset for the Nordic BCP-ALL cohort (n = 1,231).

File name - **Supplementary Data S3.**

File description - Pearson's correlation coefficient (rho) and FDR for each cross-modal element (CME) vs clinical variables, treatment protocols, clinical risk groups, and BCP ALL Subtypes.

File name - **Supplementary Data S4.**

File description - Total number of features per modality and weight after retaining features with abs(weight)>0.6.

File name - **Supplementary Data S5.**

File description - Top genes with abs(weight) >0.6 per cross-modal element (CME). The weight column (scaled from -1 to 1) denotes the impact of feature on a CME, which can be either positive or negative.

File name - **Supplementary Data S6.**

File description - Top CpG sites with abs(weight) >0.6 per cross-modal element (CME). The weight column (scaled from -1 to 1) denotes the impact of feature on a CME, which can be either positive or negative.

File name - **Supplementary Data S7.**

File description - Overlapping genes and annotated CpG sites for the same cross-modal element (CME).

File name - **Supplementary Data S8.**

File description - Significantly enriched pathways (FDR <0.01) within each CME and data modality (genes or annotated CpG sites).

File name - **Supplementary Data S9**.

File description - Top drugs with abs(weight) >0.6 per cross-modal element (CME). The weight column (scaled from -1 to 1) denotes the impact of feature on a CME, which can be either positive or negative.

File name - **Supplementary Data S10.**

File description - Network analysis per cross-modal element (CME) and weight (positive/negative) for features passing the absolute Pearson's correlation coefficient cut-off on the train set (n = 923).

File name - **Supplementary Data S11.**

File description - Network overview per cross-modal element (CME) and weight (positive/negative) for the train set (n = 923). The hub features are defined as the ones with more than 3 connections that meet the absolute Pearson's correlation cut-off criteria.

File name - **Supplementary Data S12.**

File description - Network analysis per cross-modal element (CME) and weight (positive/negative) for features passing the absolute Pearson's correlation coefficient cut-off on the test set (n = 308).

File name - **Supplementary Data S13.**

File description - Network overview per cross-modal element (CME) and weight (positive/negative) for the test set (n = 308). The hub features are defined as the ones with more than 3 connections that meet the absolute Pearson's correlation cut-off criteria.

File name - **Supplementary Data S14.**

File description - Model performance metrics, including Harrell's concordance index (mean, standard deviation, and 95% CI), were computed using stratified repeated K-fold cross-validation (5 folds × 3 repeats = 15 folds). Wilcoxon signed-rank tests were applied to compare model configurations pairwise across the 15 validation scores. P-values were adjusted using the Benjamini-Hochberg (BH) procedure to control the false discovery rate (FDR).

File name - **Supplementary Data S15.**

File description - *Ex vivo* drug response data. Fluorescence microculture cytotoxicity assay data for 857 BCP-ALL patients across 10 treatment compounds. Survival index (SI%) represents the %age of surviving cells after 72h exposure to each of these drugs.

File name - **Supplementary Data S16.**

File description – Source data for main figures Fig.1-6
